# Are Work Demand, Support and Control Associated with Work Ability and Disability during Back Pain Treatment? A Prospective Explorative Study

**DOI:** 10.3390/ijerph19063154

**Published:** 2022-03-08

**Authors:** Monica Unsgaard-Tøndel, Anne Lovise Nordstoga

**Affiliations:** 1 Department of Neuromedicine and Movement Science, Faculty of Medicine and Health Sciences, Norwegian University of Science and Technology NTNU, N-7491 Trondheim, Norway; 2 Department of Public Health and Nursing, Faculty of Medicine and Health Sciences, Norwegian University of Science and Technology NTNU, N-7491 Trondheim, Norway; anne.l.nordstoga@ntnu.no; 3 Trondheim Municipality, Department of Physiotherapy, N-7004 Trondheim, Norway; 4 Department of Physical Medicine and Rehabilitation, St Olavs Hospital, Trondheim University Hospital, N-7006 Trondheim, Norway

**Keywords:** low back pain, disability evaluation, primary health care, work characteristics

## Abstract

Background: Low back pain is a multifactorial disease with consequences for work ability and social participation. Improved integration of the work domain in health care management is needed. The aim of this study was to explore the relation between working conditions with outcome of low back pain treatment. Methods: Observational study of 41 patients attending physiotherapy for low back pain. Work demands, support and control were registered at baseline and work ability and disability also at baseline, with follow up after three and nine months. We used mixed-effects models to estimate the longitudinal associations between working conditions and outcome. Results: Higher work demands were related to reduced work ability (−1.1 points, 95% CI: −2.1 to −0.1) and slightly increased disability (5.6 points, 95% CI: 0.5 to 10.7). Lack of social support from colleagues was associated with reduced work ability (−2.7 points, 95% CI: −0.2 to 1.5) and disability (14.0 points, 95% CI: 4.9 to 23.1). Conclusions: This explorative study found associations between work demands and support, and work ability and disability outcome. Screening for psychosocial working conditions may influence the work ability and disability treatment outcome. The results need replication in larger samples and may indicate that patients seeking primary care management for low back pain should be screened for work demands, support and control.

## 1. Introduction

Low back pain is a public health problem and a main cause of years lived with disability worldwide [1]. It is connected to reduced work ability [2], including absenteeism [3] as well as presenteeism [4]. Persons with low back pain should be encouraged to continue working or return to work as soon as possible [5]. To achieve this, improved integration of social and environmental factors in health care management is needed [5,6]. Patients also want workplace accommodations and their employers to be informed about low back pain [7]. Nevertheless, a systematic review of prognostic factors for return to work concluded that the psychosocial work environment is understudied for persons with low back pain [8]. Implementing validated tools to promote a therapeutic dialogue and attention around psychosocial barriers may be part of the solution [9].

Work ability is complex and distinct subgroups have been identified among persons with musculoskeletal complaints and decreased work ability [10]. The updated work ability house model suggests that though work ability depends on a complex balance between environmental and individual factors, psychosocial work-related factors seem to have the strongest impact on work ability [11]. Work demand, support and control are potentially modifiable working conditions assessed by the Swedish Demand Support Control questionnaire [12]. These work-related factors have been associated with risk for coronary heart disease [13] and depressive symptoms [14]. Additionally, a prospective study of a general working population indicated a higher risk of sickness absence with increasing job demands and a lower risk of sickness absence with increasing job control and support, as reported by the simplified Demand Support Control questionnaire [15]. In line with this, a recent review found substantial evidence that psychosocial work factors, such as high workload, low job control and low social support, increase the risk of developing chronic low back pain [16]. Our point of departure was to explore the possible connection between self-reported working conditions and work ability among persons in treatment for low back pain. Health care practitioners need tools that specifically address potential work-related barriers for recovery. Based on this, we hypothesized that the Demand Support Control questionnaire could provide prognostic information in patients seeking physiotherapy for low back pain.

Given this background, we aimed to prospectively explore the relation between demands, support and control at work, and self-reported work ability and disability in patients attending physiotherapy for low back pain.

## 2. Materials and Methods

### 2.1. Study Population

The study population in the current study is based on a longitudinal observational study of patients with non-specific low back pain receiving primary care physiotherapy in the period from May 2014 and March 2017 [17]. Inclusion criteria for participating in this study were current non-specific low back pain as the primary complaint and age above 16 years. We excluded participants that were unable to read or understand Norwegian language, had severe neurologic signs, were pregnant, had recent back surgery, and who were not working (i.e., reported to be retired or students). Eligible patients were invited to the study by their physiotherapists at the first time of contact. The baseline data collection was performed before the first consultation with the physiotherapist, and follow-up measures was performed at approximately three and nine months. The study was approved by the Reginal Committee for Ethics in Medical Research.

### 2.2. Outcome Variables

The primary outcome was self-rated work ability, measured by a single-item question obtained from the Work Ability Index [18]: ‘Describe your current work ability compared with the lifetime best’. The score ranges from 0 to 10, where 0 represents ‘completely unable to work’ and 10 represents ‘work ability at its best’. The secondary outcome was disability measured by the Oswestry Disability Index (ODI) [19]. The disability score ranges from 0 to 100, where 0 represents ‘not disabled at all’ and 100 represents ‘completely disabled’.

### 2.3. Exposure Variables: Demands, Support and Control at Work

The psychosocial working conditions were measured at baseline by the simplified Demand Support Control questionnaire [12,15], consisting of 3 items for each dimension (i.e., demands, support and control). Additionally, we included one question about bullying/harassment [15]. The questions had four response alternatives (‘agree’, ‘mostly agree’, ‘don’t agree’ and ‘mostly don’t agree’ for the support dimension; and ‘yes, often’, ‘sometimes’, ‘seldom’ and ‘almost never’ for the control and demands dimension). The response on the single items were dichotomized by collapsing the two first and the two last response options.

To construct the sum scores for each of the demand, support and control dimensions, the scores for the single items were reversed, summarized, and divided by the number of items answered under each dimension. The sum score ranged from zero (‘low’) to three (‘high’).

### 2.4. Baseline Variables

Age (years), gender, body height (cm) and weight (kg), education, work status, work demands and average pain the last two weeks were obtained by questionnaire. Body mass index was calculated by dividing the body mass on the square of the body height (kg/m^2^). Level of education was categorized as ‘primary school’, ‘high school’, ‘college ≤4 years’ and ‘college >4 years’. Work status was determined by the question, ‘What is your current work status?’, with the possible response options: ‘paid work’, ‘unpaid work’, ‘retired’, ‘unemployed’, ‘student’, ‘sick leave’ or ‘disability pension’. Given the purpose of this study, we excluded the patients that reported to be retired or students. Work demands were categorized as ‘mostly sedentary’, ‘much walking’, ‘much walking and lifting’ and ‘heavy physical work’.

### 2.5. Statistical Analyses

Repeated measurements were analyzed by using linear mixed-effects models with a random intercept to estimate the change in the outcome variables from baseline to three- and nine-months follow-up, and the longitudinal associations between work demands, support and control at baseline and the outcomes measured at baseline, three months and nine months. The mixed-effects model accounts for dependency of observations within persons and utilize all available data irrespective of missing data at some time points. The estimates are interpreted as changes in the outcome variable per unit increase of the explanatory variables. The normality assumptions were assessed by histogram and QQ plots. All associations were adjusted for gender, age, education, average pain intensity and work demands. Precision of the associations was assessed by a 95% confidence interval. The analyses were performed using Stata version 16.1.

## 3. Results

Patient characteristics are presented in Table 1. A total of 41 participants participated in this study; 37 participants completed the three months follow-up and 27 completed the nine months follow-up. Mean age was 42.4 years (SD = 13.6), and 25 (61%) were female. Five of the patients were on sick leave at baseline (12%). The baseline average scores of the support, demands and control dimensions were 2.5 points (0.6), 2.2 points (0.6) and 1.8 points (0.6) on a 0–3 (0 = low, 3 = high) ordinal scale, respectively. Table 2 presents the mean change in work ability and disability from baseline to three- and nine-months follow-up. For both outcome variables, the change mainly occurred from baseline to three months: an increase in work ability by 1.6 points (95% CI: 1.0 to 2.1) and a decrease in disability by −9.6 points (95% CI: −12.6 to −6.6).

Table 3 shows the sum score of each dimension and associations with work ability and disability, and Table 4 shows the single-item association with work ability and disability within all dimensions.

Work demands: A higher work demands sum score was associated with reduced work ability (−1.1 points, 95% CI: −2.1 to −0.1) and slightly increased disability (5.6 points, 95% CI: 0.5 to 10.7) (Table 3). For the single items of work demands, people not reporting working effort to be too high have a higher work ability (1.2 points, 95 % CI: 0.1 to 2.2) compared to people reporting that their job requires too great work effort (Table 4).

Work support: The average sum of the scores on the support dimension were weakly associated with disability (−4.2 points, 95 % CI: −8.4 to 0.1). For the single-item reporting, a lack of support from coworkers was associated with a reduced work ability (−2.7 points, 95% CI: −4.5 to −0.9) and increased disability (14.0 points, 95% CI: 2.9 to 23.1) compared to patients reporting support from coworkers. Furthermore, not getting along with coworkers were associated with increased disability (16.3 points, 95% CI: 4.9 to 27.7) compared to people reporting to get along with their coworkers.

Work control: No associations were found between the sum score of the control dimension and work ability and disability outcome during follow-up. For the single item on the possibility to decide for yourself how to carry out your work, people reporting seldom/almost never had 1.9 points (95 % CI: −3.8 to 0.0) reduced workability compared to people reporting yes, often/sometimes. Reporting lack of possibility to decide what should be done in their work were slightly associated with increased disability (6.3 points, 95 % CI: 0.8 to 11.8) compared to people reporting often or sometimes the possibility to decide for themselves.

## 4. Discussion

In the present exploratory study, we investigated the relation between the baseline scores on the Demand Support Control questionnaire and the outcomes work ability and functional disability, as reported by patients with low back pain receiving primary care physiotherapy. Higher work demands were associated with reduced work ability and slightly increased disability. The single item of support from coworkers was also associated with both outcomes. No association between the work control and outcomes were observed in this sample.

Work ability assessment has been widely used in occupational research and has shown to be a predictor of future sickness absence [20], associated with other health-related outcomes such as pain, disability, quality of life [21], and perceived health [22], and even with disability and health 28 years after [23]. Work involving high demands of the lower back has been related to work ability among physical workers with musculoskeletal pain. Regardless of the specific type of physical work demand, being exposed to multiple physical work demands for more than a quarter of the workday was also cross-sectionally associated with lower work ability [24]. This is in accordance with the results from the present study that also indicate a relation with high work demands and inferior outcomes for work ability and disability.

Few studies have investigated the longitudinal associations between work demands, support and control and the treatment outcomes work ability and disability in primary care. A survey among physicians and patients in general practice indicated that high authority over decisions and high social support at work were related to 30% or more reduced probability for sickness certification [25]. A prospective study of patients referred to secondary care with back and or neck pain found that the perception of support and control at work were associated with disability measured by the Oswestry Disability Index or neck disability index [26]. A recent study found that low appreciation of superior and low job control was associated with depressive symptoms [27]. Our study’s results are somewhat in line with this, suggesting that aspects of work support in addition to work demands are connected to treatment outcomes, work ability and disability.

Social support at work has been related to outcome in earlier studies, though with varying populations and measurements. In the Oslo Health study, low support from superiors for women, and low job control for men, were related to sickness absence due to musculoskeletal diagnosis [28]. In another large prospective study of a general working population, low levels of supportive leadership were associated with increased risk of work disability 3 years later [29]. Worker autonomy and relationships with management has also been related to the work disability index in a cross-sectional study of coal miners [30]. Additionally, a cross-sectional study of Japanese hospital workers found that interpersonal relationships at work was associated with work-disabling low back pain [31]. Both studies indicate the importance of experiencing social support to achieve healthy work participation, and the current study expands on these previous findings by showing that lack of social support at work were associated with decreased work ability in a sample attending treatment and followed longitudinally. In the present study, one single item on social support from coworkers was related to both outcomes, though the social support dimension in sum was not.

The interpretation of results is not straightforward, and we cannot exclude the interrelatedness between the different dimensions in the Demand Support Control scale, though the three-dimensional factor structure has been confirmed empirically [12]. Resource-providing leadership has been observed to interact with, and possibly buffer, emotional demands, and to have a positive impact on employees’ work ability in female-dominated sectors [32]. The meaning of social support may be complex. One distinction can be made between directive social support, where the provider resumes responsibility, and nondirective social support, where the receiver has the control [33]. Nondirective social support has earlier been related to the perception of lower job demands and higher job control. Directive social support had the opposite relationship [33]. The interrelation between personal factors, such as attitudes and self-efficacy and the social environment, should be studied in more detail [34].

The aim of this study was to explore the potential relevance of the demand support control model in primary care management of low back pain. If the intention is to increase healthy work participation, early intervention for present workers with reduced work ability as well as absent workers is important [11]. If work-related barriers for low back pain recovery are present, the Demand Support Control Questionnaire can assist the identification of distinct areas for intervention aiming at improving the congruence between personal resources and needs, and the work environment. Individually focused interventions do not influence work ability, according to a systematic review [35]. A German study found that psychosocial working conditions at the group level accounted for a relevant proportion of sickness absence and the authors suggest that psychosocial factors are targeted at the group-level [36]. Early workplace dialogue in primary care physiotherapy improved work ability—defined as the number of sick leave days per year—in a randomized controlled trial [37]. However, self-reported work ability at 12 months was not superior in the group receiving workplace dialogue [38]. Importantly, a systematic review concluded that though work ability may be enhanced by physical activity interventions, more studies are needed on how to tailor such interventions to work demands [39]. When it comes to our secondary outcome, disability outcome was improved by workplace interventions for low back pain [40]. Work demands and control differ between different healthcare professions and interventions must be tailored to this [41]. For instance, muscular fitness and age have been related to work ability among physical therapists [42]. A qualitative study has identified age, skills and training as possible barriers for returning to work among persons with musculoskeletal pain and reduced work ability. Our results are in line with these findings by showing that screening for work demands and support were related to work ability and disability after primary care physiotherapy for low back pain. If the Demand Support Control questionnaire can be validated in clinical contexts, it may provide a tool to address potentially modifiable work-related obstacles for recovery.

A strength of the study is the prospective design with repeated measurement of the outcome variables. However, this is an explorative study aiming at hypothesis generation rather than testing. Due to the small sample size and large heterogeneity in the study population, the results will need replication in studies with larger material and further clinical validation. The content of the questionnaire and single items need further context-specific validation. Provided that our results can be replicated, in-depth investigations on the therapeutic dialogue around psychosocial working conditions will be needed.

## 5. Conclusions

Patients’ ratings of work demands and social support from colleagues were related to the work ability and disability treatment outcomes in this exploratory study.

## Figures and Tables

**Table 1:** Patient characteristics and psychosocial work conditions at baseline.

Characteristics	Baseline
Female/male, *n* (%)	25 (61)/16 (39)
Age, mean (SD)	42.4 (13.6)
BMI, mean (SD)	25.1 (4.4)
Education < = 13 years, *n* (%)	21 (51%)
On sick leave, *n* (%)	5 (12%)
Average pain last two weeks (0–10), mean (SD)	5.3 (2.2)
Work support (0–3 ^a^), mean (SD)	2.5 (0.6)
Work control (0–3 ^a^), mean (SD)	2.2 (0.6)
Work demands (0–3 ^a^), mean (SD)	1.8 (0.6)
Often/sometimes bullying/harassment at work, *n* (%)	4 (10)

Abbreviations: SD = standard deviation; BMI = body mass index. ^a^ 0 = ‘low’, 3 = ‘high’ for all dimensions.

**Table 2:** Baseline values and estimated mean change of the outcome measures work ability and disability from the baseline values to three- and nine-months follow-up.

	Baseline Mean (SD)	Mean (95% CI) Change at 3 Months	Mean (95% CI) Change at 9 Months
Work ability, 0–10 ^a^	6.3 (2.3)	1.6 (1.0 to 2.1)	1.8 (1.2 to 2.5)
Disability, 0–100 ^b^	23.0 (12.4)	−9.6 (−12.6 to −6.6)	−10.3 (−13.6 to −7.0)

Abbreviations: SD = standard deviation; CI = confidence interval. ^a^ 0 = ‘completely unable to work’, and 10 = ‘work ability at its best’. ^b^ Measured by Oswestry Disability Index: 0 = ‘not disabled at all’, and 100 = ‘completely disabled’.

**Table 3:** Associations between the mean scores of the demands, support and control dimensions at work and work ability and disability during physiotherapy treatment (measured at baseline, 3 and 9 months). Estimates reflect changes in the outcome variable per unit increase of the demand, support and control variables.

		Estimated Change in Work Ability ^a^		Estimated Change in Disability ^b^
	Crude	Adjusted Mean (95% CI)	Crude	Adjusted Mean (95% CI)
Demands ^c^	−1.1	−1.1 (−2.1 to −0.1)	6.3	5.6 (0.5 to 10.7)
Support ^c^	1.0	0.6 (−0.2 to 1.5)	−7.7	−4.2 (−8.4 to 0.1)
Control ^c^	0.4	0.3 (−0.8 to 1.5)	−6.0	−4.6 (−10.2 to 0.9)

Abbreviations: CI = confidence interval. Estimated mean change was adjusted for gender, age, education, average pain intensity and work demands (mostly sedentary, much walking, much walking and lifting, heavy physical work). ^a^ 0 = ‘completely unable to work’, 10 = ‘work ability at its best’. ^b^ Measured by Oswestry Disability Index: 0 = ‘Not disabled at all’ and 100 = ‘completely disabled’. ^c^ Measured by simplified Demand Support Control questionnaire. Average score of the three single items within each dimension.

**Table 4:** Association between work environment (single items of Demand Support Control questionnaire) and work ability and disability measured at baseline, three and nine months for patients receiving physiotherapy treatment. Estimates reflect the mean change in the outcome variables per unit change (category change) in the demand, support and control variables.

	Estimated Change Work Ability ^a^		Estimated Change Disability ^b^	
	Crude	Adjusted Mean (95% CI)	Crude	Adjusted Mean (95% CI)
Demands				
Does your job require you to work very fast? *Yes, often/sometimes* vs. *Seldom/almost never*	−0.1	−0.1 (−1.4 to 1.2)	−2.0	−0.4 (−6.6 to 5.8)
Does your job require you to work very hard? *Yes, often/sometimes* vs. *Seldom/almost never*	−0.3	−0.3 (−1.3 to 0.8)	0.0	−3.8 (−8.7 to 1.1)
Does your job require too great a work effort? *Yes, often/sometimes* vs. *Seldom/almost never*	1.5	1.2 (0.1 to 2.2)	−3.3	2.8 (−0.9 to 6.5)
**Support**				
There is good collegiality at work *Agree/mostly agree* vs. *Don’t agree/mostly don’t agree*	1.1	0.3 (−0.8 to 1.3)	−3.2	−0.9 (−5.9 to 4.1)
My coworkers are there for me (support me) *Agree/mostly agree* vs. *Don’t agree/mostly don’t agree*	−3.2	−2.7 (−4.5 to −0.9)	23.5	14.0 (4.9 to 23.1)
I get along well with my coworkers *Agree/mostly agree* vs. *Don’t agree/mostly don’t agree*	−3.0	−1.8 (−4.1 to 0.6)	29.4	16.3 (4.9 to 27.7)
**Control**				
Does your job require creativity? *Yes, often/sometimes* vs. *Seldom/almost never*	1.8	2.4 (−0.1 to 4.9)	1.0	−1.6 (−14.6 to 11.4)
Do you have the possibility to decide for yourself how to carry out your work? *Yes, often/sometimes* vs. *Seldom/almost never*	−2.2	−1.9 (−3.8 to 0.0)	12.3	7.6 (−1.9 to 17.1)
Do you have the possibility to decide for yourself what should be done in your work? *Yes, often/sometimes* vs. *Seldom/almost never*	−0.7	−0.8 (−2.0 to 0.4)	7.2	6.3 (0.8 to 11.8)
**Bullying/Harassment**				
Are you bullied/harassed at work? *Yes, often/sometimes* vs. *Seldom/almost never*	1.0	0.3 to (−1.5 to 2.0)	−15.3	−7.5 (−15.6 to 0.5)

Abbreviation: CI = confidence interval. Estimated mean change were adjusted for gender, age, education, average pain intensity and work demands (mostly sedentary, much walking, much walking and lifting, heavy physical work). ^a^ 0 = ‘completely unable to work’, and 10 = ‘work ability at its best’. ^b^ Measured by Oswestry Disability Index: 0 = ‘not disabled at all’, and 100 = ‘completely disabled’.

## Data Availability

No additional data available.
